# Brain Metabolite Changes in Patients with Relapsing-Remitting and Secondary Progressive Multiple Sclerosis: A Two-Year Follow-Up Study

**DOI:** 10.1371/journal.pone.0162583

**Published:** 2016-09-16

**Authors:** Dorothea Obert, Gunther Helms, Muriel B. Sättler, Klaus Jung, Benedikt Kretzschmar, Mathias Bähr, Peter Dechent, Ricarda Diem, Katharina Hein

**Affiliations:** 1 Department of Neurology, University Medical Center Göttingen, Göttingen, Germany; 2 Department of Cognitive Neurology, University Medical Center Göttingen, Göttingen, Germany; 3 Department of Medical Statistics, University Medical Center Göttingen, Göttingen, Germany; 4 Institute for Animal Breeding and Genetics, University of Veterinary Medicine Hannover, Hannover, Germany; 5 Department of Neurology, University Clinic Heidelberg, Heidelberg, Germany; JAPAN

## Abstract

Magnetic resonance spectroscopy (MRS) provides the unique ability to monitor several disease-related pathological processes via their characteristic metabolic markers *in vivo*. In the present study metabolic compositions were assessed every six months over the period of two years in 36 patients with Multiple Sclerosis (MS) including 21 relapsing-remitting (RR), 15 secondary progressive (SP) patients and 12 normal subjects. The concentrations of the main MRS-detectable metabolites *N-*acetylaspartate and *N-*acetylaspartylglutamate (tNAA), creatine and phosphocreatine (tCr), choline containing compounds (Cho), *myo*-Inositol (Ins), glutamine and glutamate (Glx) and their ratios were calculated in the normal appearing white matter (NAWM) and in selected non-enhancing white matter (WM) lesions. Association between metabolic concentrations in the NAWM and disability were investigated. Concentration of tNAA, a marker for neuroaxonal integrity, did not show any difference between the investigated groups. However, the patients with SPMS showed significant reduction of tNAA in the NAWM over the investigation period of two years indicating diffuse neuroaxonal loss during the disease course. Furthermore, we found a significant increase of Ins, Ins/tCr and Ins/tNAA in WM lesions independently from the course of the disease suggesting ongoing astrogliosis in silent-appearing WM lesions. Analyzing correlations between MRS metabolites in the NAWM and patients clinical status we found the positive correlation of Ins/tNAA with disability in patients with RRMS. In SPMS positive correlation of Cho with disability was found.

## Introduction

Multiple Sclerosis (MS) is a chronic inflammatory, demyelinating and degenerative autoimmune disorder of the central nervous system (CNS) that is characterized by unpredictable clinical relapses and remissions, and by progression of disability over time [1]. The detailed knowledge of the complex pathological processes of MS including reactive astrogliosis, oligodendroglial loss, axonal damage and remyelination comes from histopathological studies on biopsy and postmortem samples. However, the correlation of clinical presentation and pathological changes is extremely difficult due to the limited availability of well-characterized tissue from MS-patients. Moreover, the longitudinal observation of disease evolution in the same patient by histopathology is not possible. Conventional magnetic resonance imaging (MRI) is a routine clinical procedure for diagnosis and therapeutic follow-up of MS patients, but its pathological specificity to distinguish inflammation from demyelination, axonal loss or gliosis is limited [2, 3]. Also MRI is limited in detection of subtle, disease-related changes in the normal-appearing white matter (NAWM) [4]. These limitations can be overcome to some extent by combining MRI with MR spectroscopy (MRS). MRS offers the ability to monitor several pathological processes via their characteristic metabolic markers: *N-*acetylaspartate and *N-*acetylaspartylglutamate (tNAA) for neuroaxonal integrity, creatine and phosphocreatine (tCr) as a putative marker for cell proliferation, choline containing compounds (Cho) involved in membrane turnover, e.g. in the course of inflammation, demyelination and remyelination, *myo*-Inositol (Ins) for glial (astrocytic) proliferation, glutamate and glutamine (Glx) representing glia-neuron metabolism and lactate (Lac) indicating nonoxidative glucose consumption [2, 5–10]. Abnormal concentrations of these metabolites have been found in the brain and spinal cord of MS-patients when compared with controls [10, 11]. Furthermore, MRS-studies found an association of metabolites level with cognitive impairment [11, 12] and clinical disability measured by the Expanded Disability Status Scale (EDSS) [13, 14]. In particular, based on histopathological and imaging investigations tNAA has been shown to be the most important substrate for axonal pathology and MS-related disability [14, 15]. More recent MRS-studies focused on investigation of the metabolite ratios with the ultimate goal of providing more specific insights into pathological processes involved in the disease evolution of MS. Here, tNAA/Cho has been found to constitute as an efficient marker to differentiate progressive forms of MS from controls [15, 16] and from relapsing remitting MS (RRMS) [16, 17]. Additionally, changes in Ins/tNAA in NAWM were found to have predictive power on brain atrophy and neurological disability evolution [17, 18].

In our study, we performed serial measurements of metabolites concentrations in patients with RRMS, secondary progressive MS (SPMS) and controls over the period of two years. With regard to the relevance of tNAA for clinical disability, we firstly proposed to identify the stage of the disease with the most prominent tNAA reduction indicating a critical time-point of irreversible disability. We hypothesized that patients with SPMS would show lower tNAA level. Secondly, correlations between MRS metabolites and patients clinical status were analyzed to better understand the underlying pathological processes and their clinical expression.

## Material and Methods

### Study population

**Fig 1:**
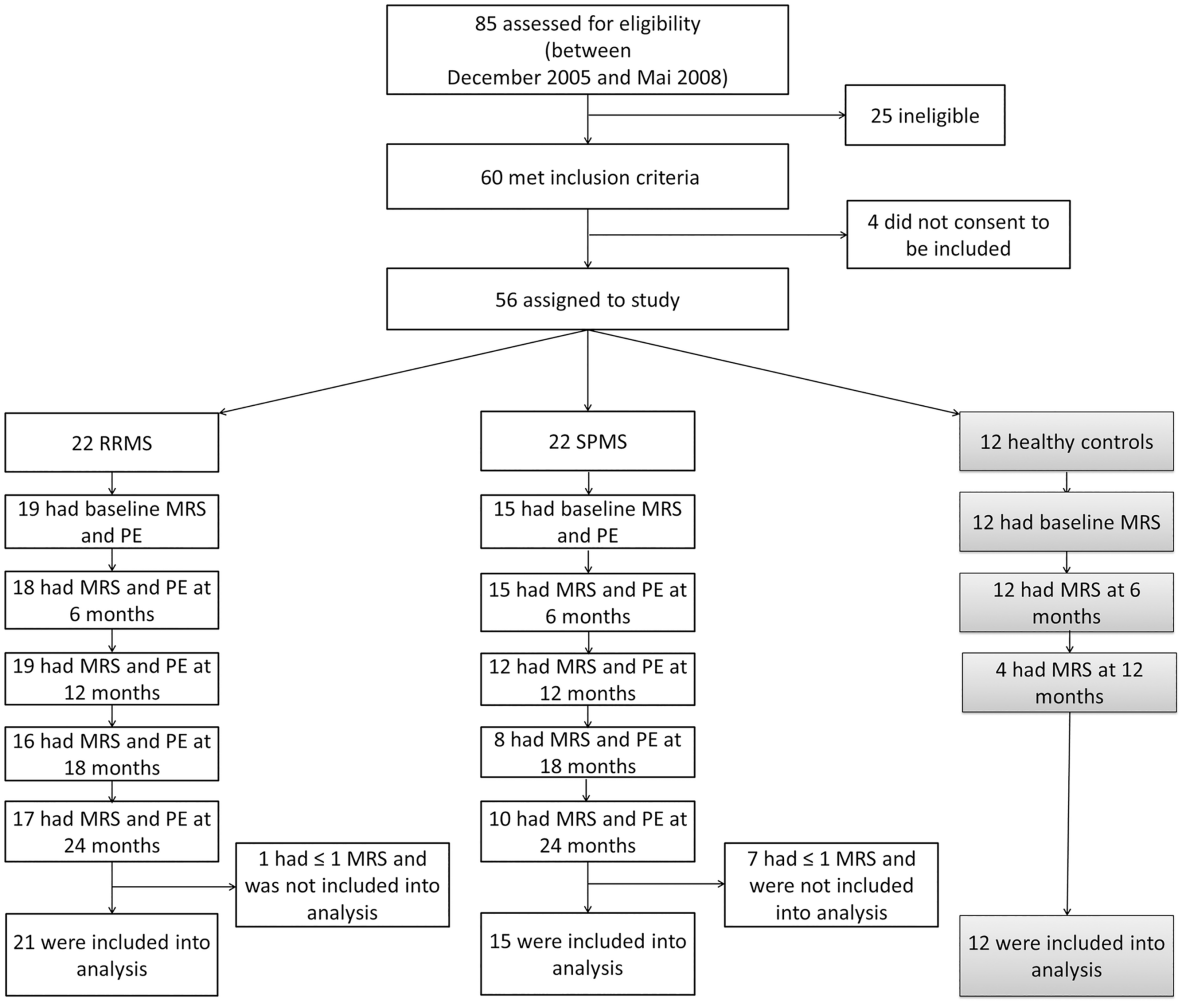
Flowchart for the study procedure. Final patient numbers correspond to those included in the analysis. MRS: Magnetic resonance spectroscopy; PE: physical exam.

Fourty-four (44) patients with clinically definite MS between 18–60 years of age were chosen from the population followed in the outpatient MS clinic, Department of Neurology, Göttingen, Germany, for this parallel group observation (Fig 1). Patients were classified according to clinical course as having either recurrent relapses with at least one clinical relapse within the last 12 months and noticeable residual symptoms (n = 22) or secondary progressive disease course over a maximum period of two years prior to inclusion in the absence of even discrete relapses after earlier relapsing-remitting disease course (n = 22). Exclusion criteria consisted of any contra-indications against the MRI-scan (e.g. cardiac pacemaker, metal implants, gadolinium allergy). Patients were entered into the study only if they had been free from attacks in the previous eight weeks. The rationale for this selected population was to reduce the potential confounding of reversible NAA changes occurring after acute relapse [18, 19]. If a patient had an acute clinical relapse during the follow up, MRS was postponed for eight weeks. Twelve healthy age- and sex-matched volunteers were included in this study as a control group. Patients were observed over a period of 2 years. Every 6 months MRS of NAWM and white matter lesions were performed at the Department of Cognitive Neurology, University Medical Center Göttingen, Germany (Fig 1).

For purpose of anonymization and blinding, patient exams were registered by a code number; image data were stored on a RAID system. Three spectroscopies were planned for healthy controls, since no significant change was being expected over the period of two years. When the first 4 healthy controls had undergone 3 MRS we did not see any significant changes in the metabolites over the time. Because of that and due to reasons of capacity the number of healthy controls was reduced to 12 (20 were originally planned) and the third MRS was cancelled in the other 8 healthy controls. Eight patients dropped out after the first examination. In ten patients, measurement of NAWM was not possible since NAWM could not be identified due to high lesion load. Therefore in these cases only MRS of the lesions was enrolled into the analysis. In 2 patients NAWM changed into a lesion during observation period. In those two cases only MRS of NAWM was enrolled into analysis. Patients with two or more spectroscopies were included into analyses, leaving 21 RRMS, 15 SPMS and 12 healthy controls. Beside MRS, patients underwent a full neurological examination by a neurologist every 6 months to determine the disability by EDSS and Multiple Sclerosis Functional Composite (MSFC). Data were collected in a standardized electronic chart (Multiple Sclerosis Documentation System (MSDS), Version 3.0). The local ethical committee of the University of Göttingen approved the study in 2004 (Reg.- nr.: 12/10/04). Registration in a clinical trial registry was not required by the local ethic committee due to its observational nature. Retrospective registration was done in German registry for clinical studies (www.drks.de; Reg.-nr. DRKS00009807). The authors confirm that all ongoing and related trials for this drug/intervention are registered. Written informed consent was obtained from all participating subjects prior to study enrollment and prior to each biannual examination.

### MRS protocol

The study was performed on a 3T whole-body MRI system (Magnetom Trio, Siemens, Germany) using an 8-channel receive-only head coil and the body coil for transmission. MRI was performed at 1 mm isotropic resolution. For T1-weighting, three dimensional magnetization-prepared rapid acquisition gradient echo imaging was applied (MP-RAGE, 176 non-selective sagittal partitions, inversion time TI = 900 ms, repetition time TR = 2250 ms, flip angle FA = 9°, bandwidth BW = 200 Hz/pixel, echo time TE = 3.26 ms). For T2-weighting, a turbo spin echo sequence with variable refocusing flip angles was used (TSE, 144 partitions across an axial slab, TR = 2900 ms, echo train length 808, effective TE = 419 ms, BW = 434 Hz/pixel, parallel imaging with acceleration factor 2, 2 avarages). Furthermore, a TSE sequence with fluid attenuation by inversion recovery (FLAIR) contrast was acquired (176 non-selective sagittal partitions, TI = 2100 ms, TR = 6000 ms, echo train length 246, effective TE = 403 ms, BW = 698 Hz/pixel, parallel imaging with acceleration factor 2). MP-RAGE was re-performed after intraveneous administration of a gadolinium-based contrast agent (single dose of 0.1 mmol per kg body weight, Gadovist, Bayer Vital GmbH, Leverkusen, Germany) to identify acute inflammatory lesions.

**Fig 2:**
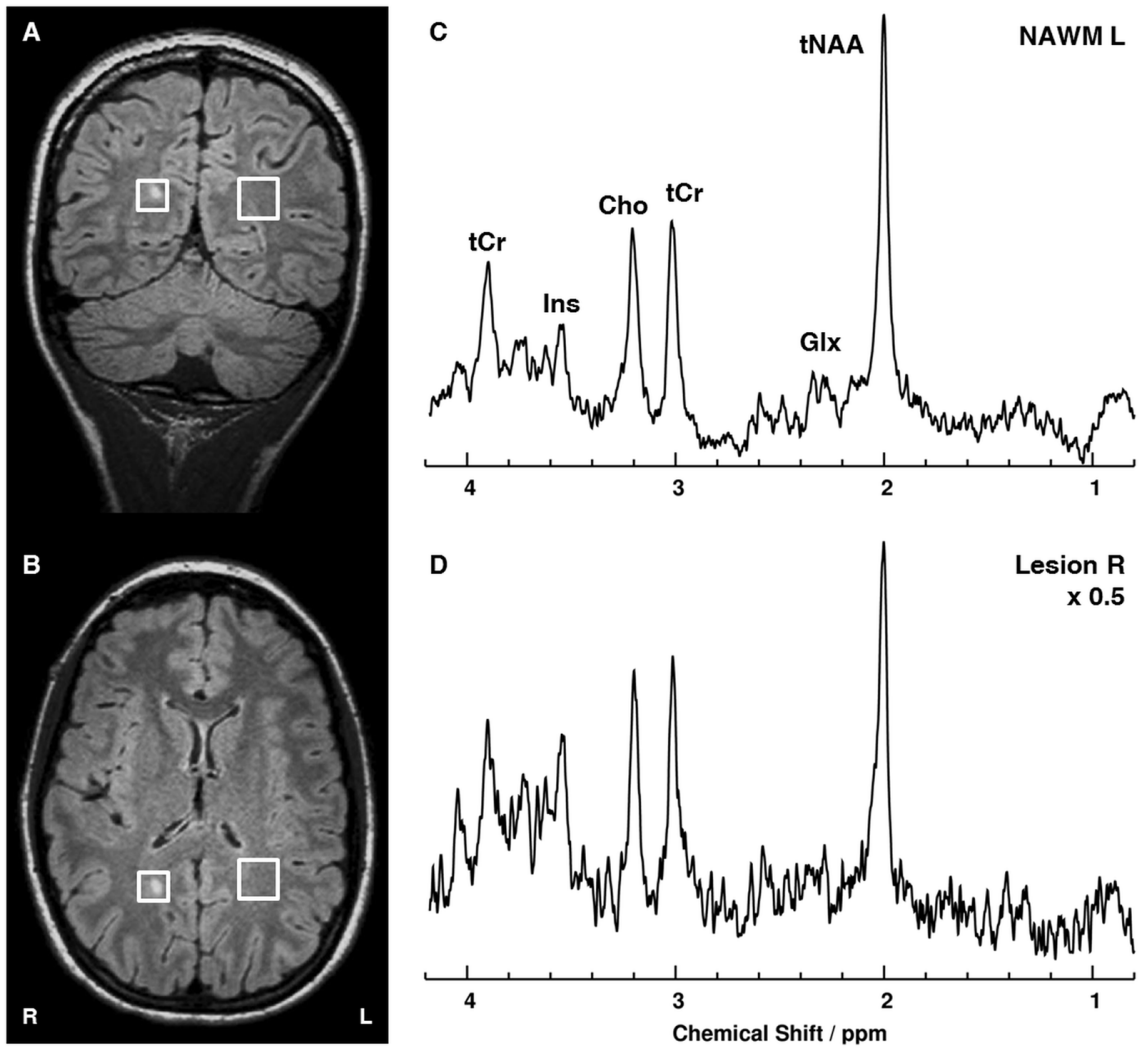
(A) Coronal and (B) axial MR images with FLAIR contrast of a patient with relapsing-remitting Multiple Sclerosis as well as fully relaxed short-echo time proton MR spectra from (C) left normal appearing white matter (NAWM L; 4.1 ml) and (D) a right white matter lesion (Lesion R; 1.7 ml). Locations for MRS are indicated by white boxes. tNAA: *N-*acetylaspartate and *N-*acetylaspartylglutamate; tCr: creatine and phosphocreatine; Cho: choline containing compounds; Ins: *myo*-Inositol; Glx: glutamate and glutamine. L: left; R: right.

For MRS, lesions of suitable size were selected inside the WM and one region of NAWM. Fully relaxed short-echo time proton MR spectra (TR/TE/mixing time TM = 6000/20/10 ms, 64 accumulations) were acquired with use of a single–voxel stimulated echo acquisition mode (STEAM) localization sequence. If possible, volumes of interest (VOI; mean ± SD = 3.06 ± 0.91 ml, min = 1.7 ml, max = 4.6 ml) were located in two different non-enhancing lesions in WM as well as in NAWM. 64 acquisitions were averaged without individual frequency correction. To reduce the contributions to lesion VOIs from surrounding tissue, the VOIs were individually adjusted in size to circumscribe the lesion (see Fig 2).This minimized adjacent CSF and gray matter in the VOI and kept contributions from NAWM approximately constant, but resulted in rather small volumes. Notwithstanding the lower signal-to-noise ratio (SNR) than in NAWM (Fig 2), analysis by LCModel [20] in the 1.0–4.2 ppm range yielded concentrations estimates in millimoles/liter (mM). For main metabolites, precision of estimated concentrations was sufficient (< 20% SD) due to phase-coherent addition of multiple receive channels [21] (zero phase) and choice of short TE and long TR. SNR and spectral linewidth (FWHM: full width at half maximum), parameters reflecting spectral quality, were retrieved from LCModel and are given in the tables. Major detectable metabolites include tNAA, tCr, Cho, Ins, and Glx. The uncombined eight signals from the receive coil array were weighted and phase-coherently added by means of an uncombined water scan and scaled to transmit-receive body coil [21]. Spectral evaluation and quantification of absolute metabolite concentrations were accomplished with use of LCModel [20, 21]. Representative VOI locations in a WM lesion and NAWM as well as corresponding MR spectra are shown in Fig 2.

### Statistical Analysis

The sample size estimation was based on previous MRS-data in patients with RRMS and SPMS [22–24]. The analysis comprised the major detectable metabolites tNAA, tCr, Cho, Ins, and Glx. Lactate was not reliably detected. Metabolite concentrations were described by their mean +/- standard deviation within each combination of the experimental factors time (0, 6, 12, 18, 24 months), group (RRMS, SPMS, control) and location (NAWM and WM-lesion). The effect of time, group and location were first studied by a three-way analysis of variance (ANOVA) for repeated measures, also considering each interaction between the main effects, i.e. the full model was considered. In subsequent two-way and one-way ANOVAs, time and group effects were studied separately for each group and each point in time, respectively. For example, when the data was split by location, the two-way ANOVA included the group and time effect plus their interaction. One-way ANOVAs considered either the time or the group effect alone as well as pairwise comparisons between time points or between groups. The significance level for the three-way ANOVA was set to α = 5%. In the case of no significant effects in the three-way ANOVA, results of the subsequent lower-way ANOVAs or pairwise comparisons were only considered significant with respect to Bonferroni-Holm corrected significance levels. Correlation between motor function (Timed 25-Foot Walk (25FW) and 9-Hole Peg Test (9HPT)) and NAWM metabolic concentrations was assessed with Pearson correlation coefficient R. Correlation between EDSS and Paced Auditory Serial Addition Test (PASAT), which are both on an ordinal scale, with NAWM metabolic concentration was calculated using Kendall’s correlation coefficient tau. Resulting p-values from correlation analyses were adjusted using again the method of Bonferroni-Holm. All analyses were performed with the software R (version, 2.14, www.r.-project.org). Repeated-measures ANOVAs were modelled using the R-package “geepack”.

### Statistical power and sample size calculation

In order to obtain a rough idea about the power of the current study and the necessary sample sizes for future studies, the observed means and standard deviations from the group comparisons at time point 1 were employed (see metabolite concentration changes in the result section). For each pairwise comparison of a parameter at time point one, we determined the actual power that the observed group difference was called significant. The mean power obtained in this calculation was 33% (minimum: 7%, median: 22%, maximum: 89%). According to our further power calculations for future studies a sample size of n = 88 per group was necessary to call the same group differences significant with a fix power of 80%. Power and sample size calculations were done using the R-package “powerAnalysis”.

## Results

### Clinical features

Patients were recruited from December 2005 until Mai 2008. Last patient completed MRI-Scan and follow up clinical examination in Mai 2010. 21 patients with RRMS (13 women, 8 men), mean age 38.8 ± 11.2 years, 15 patients with SPMS (12 women, 3 men), mean age 45.7 ± 11.9 years and 12 healthy control subjects (8 women, 4 men) mean age 42.1 ± 11.6 (range 23.2–58.1) years were enrolled into the study. Among the three different groups, there were no significant differences regarding gender (Fisher’s exact test: p = 0.53) and age (1-way ANOVA: p = 0.22). At the time of inclusion median EDSS was 2.5 (range 1–6.5) in RRMS and 4.0 (range 1–6.5) in SPMS patients (Table 1).

**Table 1:** Clinical characteristics of Multiple Sclerosis patients and control subjects.

Group	RRMS	SPMS	controls
n	21	15	12
Age (y)	38.8 ± 11.2	45.7 ± 11.9	42.1 ± 11.6
Sex (female/male)	13/8 (62%/38%)	12/3 (80%/20%)	8/4 (67%/33%)
EDSS (range)	2.5 (1–6.5)	4.0 (1–6.5)	N/A

y = years, EDSS = Expanded Disability Status Scale, N/A = not applicable. Age: mean ± standard deviation. EDSS: median (range).

### Metabolite concentration changes

**Fig 3:**
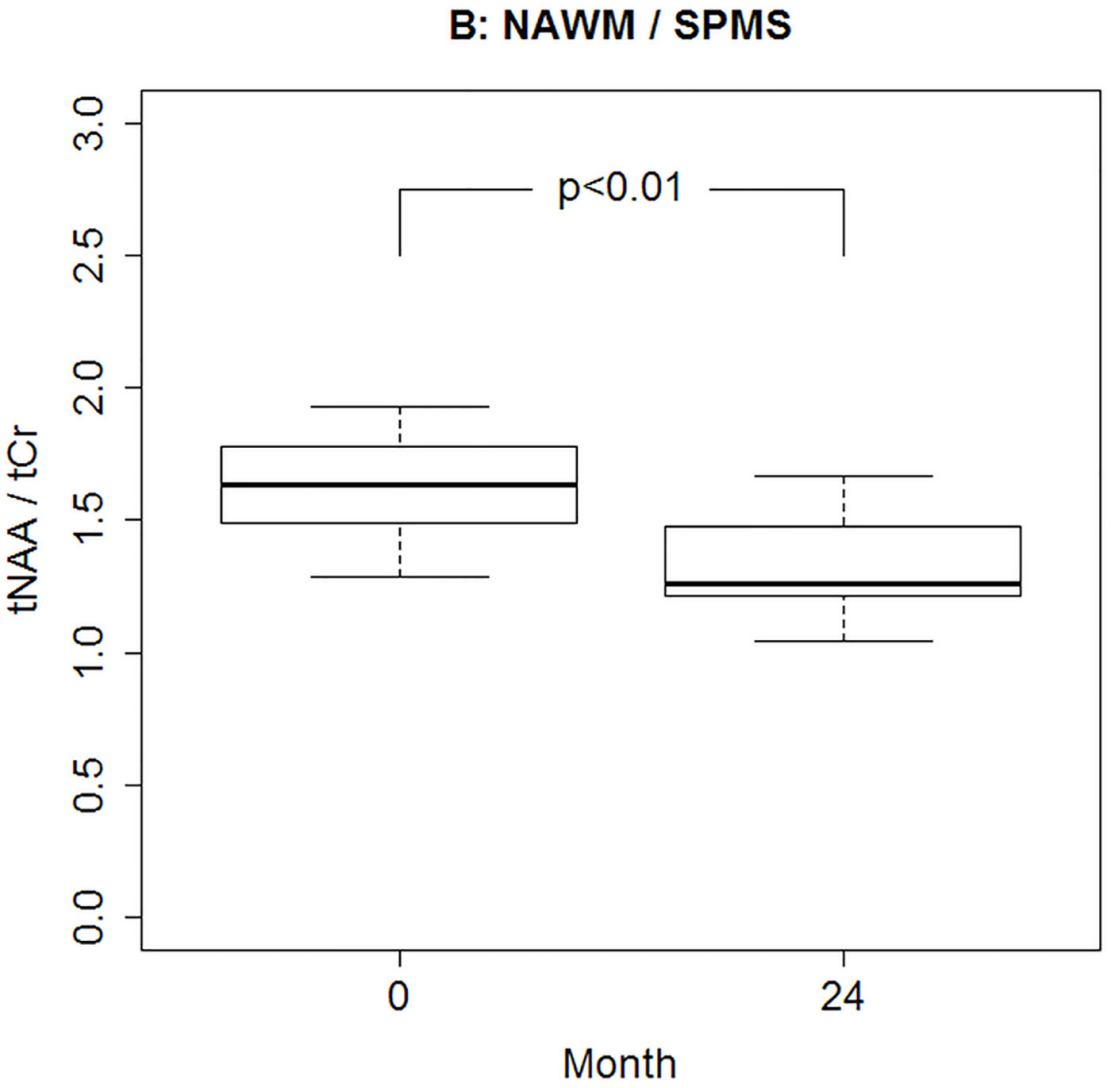
Boxpot of tNAA/tCr in patients with secondary progressive Multiple Sclerosis (SPMS) in normal appearing white matter (NAWM) at baseline (0 months) and end of study (24 months).

Mean +/- standard deviation of the metabolite concentrations are presented in Tables 2 and 3, separated by group, time and location. Analysis of metabolite concentrations as well as their ratios in NAWM and in WM-lesions revealed no significant difference between the three groups (Table 4). However, the 3-way ANOVA detected a significant time effect in tNAA/tCr (p < 0.01), that could be confirmed in 2-way ANOVAs for the NAWM (p < 0.01) as well as for the WM-lesions (p = 0.03). In the NAWM this time effect was however only present for the SPMS group (p < 0.01) due to a significant difference between time 0 and time 24 (p < 0.01, Fig 3), while only present in the RRMS group within the WM-lesions (p < 0.01). A significant interaction between time and location was observed for tNAA (p < 0.01), meaning that the overall time profile appears to be different in the NAWM and the WM-lesions. Subsequent 2-way ANOVAs yielded, however, no significant time or group effects.

**Table 2:** Mean +/- standard deviation of the individual metabolite concentrations (mM) as well as metabolite concentration ratios in the NAWM by group and time.

Parameter	Group	Time 1	Time 2	Time 3	Time 4	Time 5
tNAA	Healthy	7.95+/-0.93	7.73+/-1.28	8.22+/-1.2	NaN+/-NA	NaN+/-NA
	RR	7.69+/-1.36	7.57+/-1.11	7.71+/-1.02	6.84+/-1.2	7.23+/-1.31
	SP	7.44+/-1.19	7.37+/-0.89	7.24+/-0.92	7.38+/-1.07	6.8+/-0.88
tCr	Healthy	4.79+/-0.49	4.66+/-0.77	5.26+/-0.69	NaN+/-NA	NaN+/-NA
	RR	4.94+/-0.78	4.94+/-0.9	5.03+/-0.79	4.53+/-0.95	5.03+/-0.85
	SP	4.56+/-0.4	5.11+/-1.08	4.83+/-0.95	4.82+/-0.85	5.19+/-0.81
Cho	Healthy	1.87+/-0.28	1.9+/-0.39	1.79+/-0.2	NaN+/-NA	NaN+/-NA
	RR	1.82+/-0.41	1.81+/-0.54	1.74+/-0.23	1.6+/-0.29	1.6+/-0.26
	SP	1.63+/-0.31	1.8+/-0.23	1.69+/-0.38	1.82+/-0.33	1.83+/-0.48
Ins	Healthy	3.6+/-0.76	3.73+/-1.36	4.33+/-0.54	NaN+/-NA	NaN+/-NA
	RR	3.6+/-1.06	3.54+/-1.07	3.61+/-0.68	3.46+/-0.8	3.5+/-1.18
	SP	3.44+/-0.86	4.22+/-1.6	3.65+/-0.87	3.77+/-0.85	4.55+/-1.27
Glx	Healthy	7.14+/-1.15	7.1+/-1.86	7.89+/-0.58	NaN+/-NA	NaN+/-NA
	RR	7.73+/-1.79	7.15+/-1.91	7.31+/-1	6.65+/-1.09	7.56+/-1.78
	SP	6.98+/-1.48	7.13+/-2.22	6.25+/-1.74	8.14+/-1.78	8.58+/-1.66
Cho/tNAA	Healthy	0.24+/-0.05	0.25+/-0.04	0.22+/-0.02	NaN+/-NA	NaN+/-NA
	RR	0.24+/-0.05	0.24+/-0.05	0.23+/-0.05	0.24+/-0.04	0.23+/-0.06
	SP	0.22+/-0.04	0.25+/-0.05	0.23+/-0.05	0.25+/-0.04	0.28+/-0.1
Ins/tNAA	Healthy	0.46+/-0.13	0.48+/-0.13	0.53+/-0.09	NaN+/-NA	NaN+/-NA
	RR	0.46+/-0.12	0.47+/-0.11	0.46+/-0.12	0.53+/-0.13	0.49+/-0.18
	SP	0.47+/-0.13	0.59+/-0.26	0.51+/-0.13	0.52+/-0.13	0.66+/-0.22
tNAA/tCr	Healthy	1.67+/-0.22	1.67+/-0.18	1.59+/-0.31	NaN+/-NA	NaN+/-NA
	RR	1.57+/-0.24	1.55+/-0.22	1.55+/-0.27	1.54+/-0.25	1.47+/-0.33
	SP	1.63+/-0.21	1.5+/-0.36	1.53+/-0.27	1.56+/-0.28	1.33+/-0.24
Cho/tCr	Healthy	0.39+/-0.04	0.41+/-0.03	0.35+/-0.04	NaN+/-NA	NaN+/-NA
	RR	0.37+/-0.05	0.37+/-0.07	0.35+/-0.06	0.36+/-0.06	0.32+/-0.05
	SP	0.36+/-0.05	0.36+/-0.04	0.35+/-0.07	0.38+/-0.05	0.35+/-0.08
Ins/tCr	Healthy	0.75+/-0.11	0.79+/-0.16	0.81+/-0.07	NaN+/-NA	NaN+/-NA
	RR	0.72+/-0.16	0.72+/-0.16	0.72+/-0.09	0.79+/-0.14	0.69+/-0.17
	SP	0.75+/-0.16	0.83+/-0.28	0.76+/-0.11	0.78+/-0.12	0.8+/-0.12
Glx/tCr	Healthy	1.49+/-0.2	1.52+/-0.23	1.39+/-0.47	NaN+/-NA	NaN+/-NA
	RR	1.56+/-0.25	1.48+/-0.32	1.47+/-0.21	1.52+/-0.18	1.51+/-0.24
	SP	1.52+/-0.21	1.39+/-0.29	1.29+/-0.2	1.72+/-0.24	1.6+/-0.22
FWHM	Healthy	0.046 ± 0.005	0.047 ± 0.005	0.049 ± 0.001	NaN+/-NA	NaN+/-NA
	RR	0.051 ± 0.009	0.051 ± 0.008	0.051 ± 0.012	0.061 ± 0.014	0.050 ± 0.015
	SP	0.046 ± 0.009	0.057 ± 0.013	0.051 ± 0.008	0.047 ± 0.013	0.053 ± 0.012
SNR	Healthy	8.3 ± 3.1	8.8 ± 3.0	6.8 ± 2.4	NaN+/-NA	NaN+/-NA
	RR	9.3 ± 2.3	9.3 ± 2.4	8.6 ± 2.9	6.6 ± 2.9	6.8 ± 3.1
	SP	8.7 ± 3.0	8.1 ± 2.6	7.4 ± 1.4	8.0 ± 2.1	5.4 ± 1.7

Measures of spectral quality (FWHM: full width at half maximum (ppm); SNR: signal-to-noise ratio) are given.

**Table 3:** Mean +/- standard deviation of the individual metabolite concentrations (mM) as well as metabolite concentration ratios in the WM-lesions by group and time.

Parameter	Group	Time 1	Time 2	Time 3	Time 4	Time 5
tNAA	RR	7.39+/-0.86	7.22+/-1.02	7.3+/-1.18	7.33+/-1.13	7.84+/-1.08
	SP	6.71+/-1.24	6.61+/-1.17	6.59+/-0.96	7.11+/-1.11	7.8+/-1.81
tCr	RR	4.79+/-0.52	4.84+/-0.85	4.83+/-0.83	5.02+/-0.78	5.17+/-0.85
	SP	4.84+/-0.7	5.09+/-0.54	4.58+/-0.53	4.85+/-0.77	5.55+/-0.87
Cho	RR	1.92+/-0.39	1.78+/-0.32	1.64+/-0.28	1.94+/-1.07	1.8+/-0.41
	SP	1.81+/-0.38	1.8+/-0.3	1.76+/-0.28	1.86+/-0.46	2.04+/-0.46
Ins	RR	4.26+/-0.99	3.99+/-0.79	3.9+/-0.7	4.04+/-0.81	4.15+/-0.71
	SP	4.43+/-1.19	4.66+/-1.01	4.16+/-1.23	4.38+/-1.14	5.09+/-1.14
Glx	RR	7.51+/-1.57	7.45+/-1.4	7.34+/-1.9	7.52+/-1.78	7.25+/-1.88
	SP	7.49+/-1.45	7.06+/-1.33	6.5+/-1.21	7.33+/-2.29	8.48+/-0.97
Cho/tNAA	RR	0.26+/-0.06	0.25+/-0.04	0.23+/-0.06	0.28+/-0.2	0.23+/-0.06
	SP	0.28+/-0.06	0.28+/-0.08	0.27+/-0.04	0.26+/-0.05	0.27+/-0.09
Ins/tNAA	RR	0.59+/-0.17	0.57+/-0.13	0.56+/-0.16	0.56+/-0.12	0.53+/-0.1
	SP	0.7+/-0.23	0.77+/-0.37	0.64+/-0.22	0.61+/-0.11	0.69+/-0.22
tNAA/tCr	RR	1.56+/-0.19	1.52+/-0.25	1.54+/-0.31	1.49+/-0.31	1.54+/-0.18
	SP	1.39+/-0.19	1.31+/-0.27	1.46+/-0.19	1.48+/-0.12	1.42+/-0.3
Cho/tCr	RR	0.4+/-0.06	0.37+/-0.06	0.35+/-0.07	0.4+/-0.27	0.35+/-0.06
	SP	0.38+/-0.05	0.35+/-0.04	0.38+/-0.04	0.38+/-0.04	0.37+/-0.1
Ins/tCr	RR	0.89+/-0.2	0.84+/-0.14	0.82+/-0.15	0.81+/-0.13	0.81+/-0.14
	SP	0.93+/-0.26	0.91+/-0.2	0.91+/-0.28	0.9+/-0.2	0.91+/-0.22
Glx/tCr	RR	1.57+/-0.27	1.56+/-0.2	1.52+/-0.26	1.5+/-0.34	1.43+/-0.39
	SP	1.55+/-0.23	1.37+/-0.21	1.48+/-0.45	1.51+/-0.47	1.55+/-0.3
FWHM	RR	0.052 ± 0.010	0.051 ± 0.010	0.053 ± 0.010	0.061 ± 0.023	0.053 ± 0.014
	SP	0.048 ± 0.007	0.051 ± 0.009	0.053 ± 0.010	0.052 ± 0.010	0.052 ± 0.007
SNR	RR	7.0 ± 2.8	7.2 ± 3.2	6.6 ± 3.3	5.3 ± 2.2	4.2 ± 1.5
	SP	7.0 ± 2.9	6.7 ± 2.9	5.9 ± 2.4	6.2 ± 3.1	4.8 ± 1.3

Measures of spectral quality (FWHM: full width at half maximum (ppm); SNR: signal-to-noise ratio) are given.

**Table 4:** Results from the 3-way analysis of variance for the individual metabolites, reflecting the effects of group, time and location as well as their interactions.

Parameter	Group	Time	Location	Group x Time	Group x Location	Time x Location	Group x Time x Location
tNAA	0.12	0.85	0.41	0.34	0.73	< 0.01	0.32
tCr	0.94	0.01	0.70	0.40	0.56	0.21	0.59
Cho	0.93	0.69	0.29	0.01	0.76	0.69	0.90
Ins	0.30	0.38	< 0.01	0.22	0.55	0.96	0.90
Glx	0.80	0.95	0.86	0.06	0.70	0.92	0.46
Cho/tNAA	0.20	0.88	0.09	0.13	0.55	0.05	0.60
Ins/tNAA	0.07	0.49	< 0.01	0.96	0.32	0.18	0.48
tNAA/tCr	0.10	< 0.01	0.27	0.77	0.26	0.21	0.04
Cho/tCr	0.98	0.05	0.23	0.03	0.74	0.70	0.88
Ins/tCr	0.22	0.29	< 0.01	0.64	0.80	0.44	0.90
Glx/tCr	0.59	0.34	0.79	0.08	0.94	0.25	0.69

**Fig 4:**
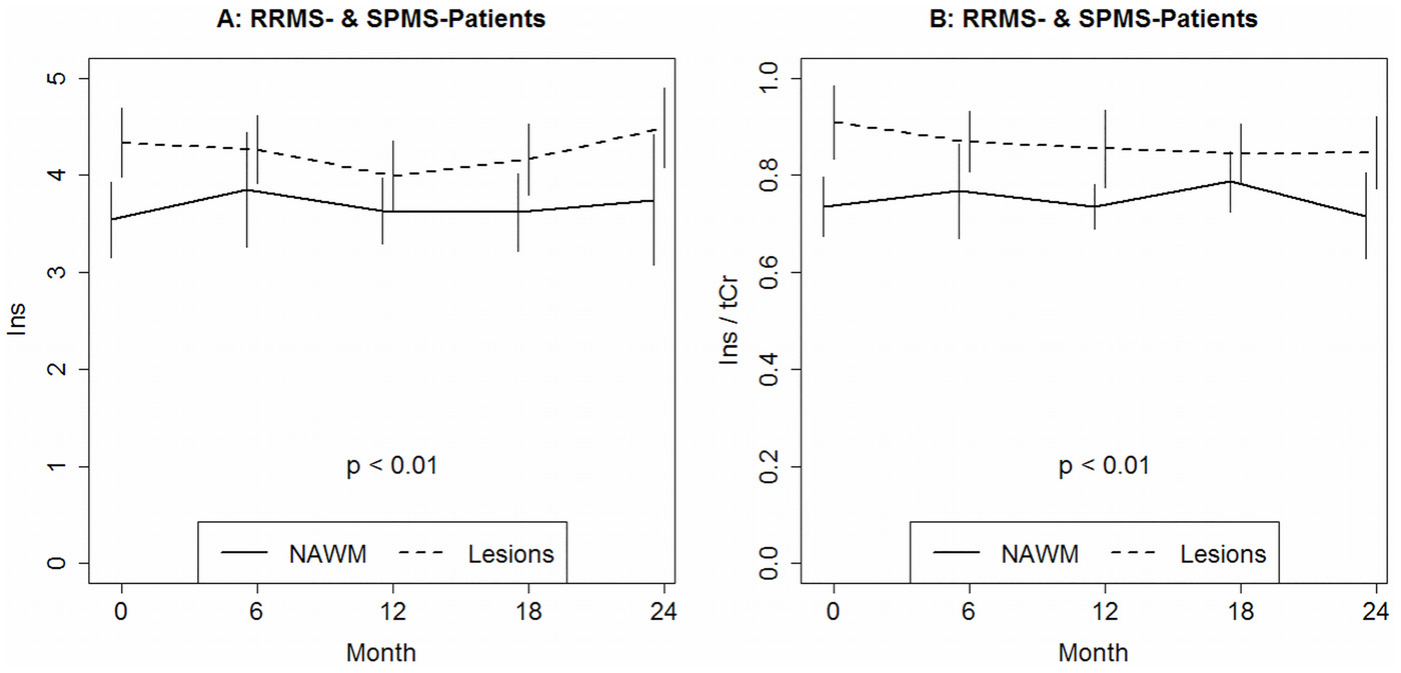
Ins (A) and Ins/tCr (B) in patients with relapsing-remitting (RRMS) and secondary progressive multiple sclerosis (SPMS) in white matter lesions (Lesions) compared to normal appearing white matter (NAWM) over two years follow up.

Results of Ins, Ins/tCr and Ins/tNAA revealed a significant location effect detected in the 3-way ANOVA (p < 0.01, Fig 4A and 4B). This effect can be explained by the fact that the overall mean in the WM-lesions was higher compared to the metabolite level detected in the NAWM. For tCr a significant effect of the time factor was observed (p = 0.01). Splitting the ANOVA by the type of location yielded that this time effect was not present in the NAWM (p = 0.37) but in the WM-lesions (p = 0.01). Subsequent analyses identified that the time effect in the WM-lesions was only present in the RRMS group (p = 0.03) but not in the SPMS group (p = 0.09). The time effect in the RRMS group could be traced back to a difference between time 0 and 24 (p = 0.03). A significant group-by-time interaction was detected for Cho and Cho/tCr by the 3-way ANOVA (p = 0.01 and p = 0.03). Subsequent analyses in the NAWM could, however, not specify this effect in greater detail. Patient age at the mid of the study interval was not used as a regressor/nuisance-variable since this would interact with the clinical groups. The observed differences, especially in the NAWM, may be partly due to normal aging.

### Correlation between metabolite concentrations in NAWM and disability

Correlation of metabolite concentration in NAWM of MS-Patients with EDSS, 9HPT, 25FW and PASAT showed many statistically significant results. Healthy controls were excluded from analysis.

**Fig 5:**
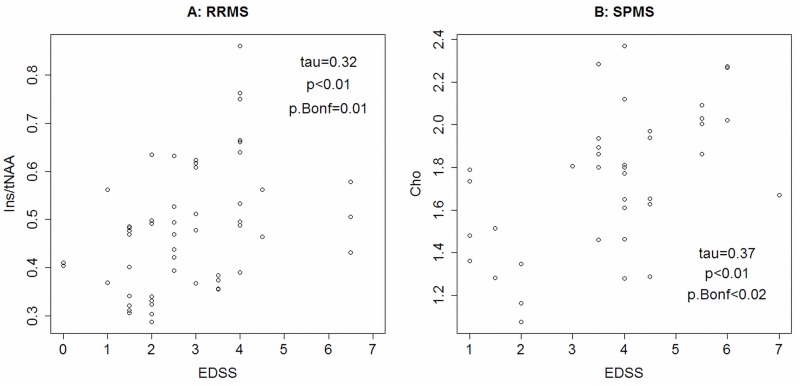
Scatter plot of Ins/tNAA and EDSS in patients with relapsing-remitting (RR) multiple sclerosis (A) and Cho and EDSS in patients with secondary progressive (SP) disease course (B).

In the subgroup analysis we found that in RRMS high levels of tNAA resulted in lower disability as evaluated by EDSS (tau = -0.22, p = 0.18) as well as in a better performance in the 25FW (R = 0.47, p = 0.01). tNAA/tCr, which is considered to be less affected by cerebral edema due to inflammation, showed the same negative correlation regarding EDSS in RRMS (tau = -0.28, p = 0.034). An increase of Ins/tNAA, reflecting gliosis and axonal loss, was associated with high EDSS (tau = 0.32, p = 0.01, Fig 5A) and poor results in 9HPT (R = 0.47, p = 0.02) in RR patients. In patients with SPMS high Cho concentrations were correlated with high EDSS (tau = 0.37, p = 0.02) and poor walking ability (R = 0.55, p = 0.01, Fig 5B).

## Discussion

In this longitudinal MRS study, metabolic concentrations were measured in lesions as well as in the NAWM of MS patients in order to identify a stage of disease with the most pronounced axonal damage and secondly, to correlate the metabolic changes with clinical disability. Patients with RR and SP-MS underwent MRS every 6 month during a two years period. Although our results confirmed the well-known MRS findings in MS (see below), we failed to confirm our main hypothesis that patients with SPMS would show lower tNAA than RRMS and healthy controls. There are several tentative explanations why we failed to observe significant changes in tNAA concentration between the groups. Decreases in NAA concentration were mostly reported in chronic and in acute MS-lesions while they remain controversial in NAWM [5]. Moreover, the significant reduction in NAA/Cr ratio observed in previous studies was, in most cases, evidently due to the increased Cr level [24, 25]. Additionally, we found a relatively large variability in tNAA concentration. The difference in mean value of tNAA concentration was much larger than the difference of metabolite concentration between groups (0.09% vs. 0.04%) that could have contributed to the lack of tNAA concentration differences. Moreover, limitation of our study is the relatively small number of patients fulfilling 2 year longitudinal study. Notably, we did not observe any significant difference in MS patients compared to the control group although the trend to the decreased level could be noticed in MS-patients. Histopathological studies have indicated that axonal damage is prominent during the early stage of the disease and decreases in extent with disease progression [26–28]. More recent study on autopsy material from MS patients and controls found that the extent of axonal injury in the later stage of the disease even decline to the disease levels that are similar with age-matched controls [28, 29]. With respect to the long duration of the disease our cohort was recruited in a highly selected manner that may explain why we did not detect any significant difference in tNAA-level between the groups. However, we found a significant decrease in tNAA and in the tNAA/tCr in NAWM in SPMS over the observation period of two years indicating ongoing diffuse axonal damage in the progressive stage of the disease.

In addition to tNAA and tCr, other MRS-detectable metabolites seem to have pathophysiological as well as clinical importance. In this context several markers of glial proliferation and membrane turnover associated with inflammation as Ins and Cho [29, 30] were measured in NAWM and in WM lesions in this study. An important observation in this study was a significant increase of Ins concentration and Ins/tCr ratio in WM lesion independently from the course of the disease although no changes in Ins level was detected in the NAWM. Previous studies reported an increase of Ins concentration in NAWM [30, 31] as well as in WM lesions [31, 32] with a more marked increase in T1 hypointense than in isointense lesions [32, 33]. Gadolinium (Gd)-enhanced MR imaging is the gold standard for detecting disease activity of MS and is associated with abnormalities in blood brain barrier. In our study metabolite level was detected in the non-enhancing lesions suggesting ongoing astrogliosis in silent-appearing WM lesions during the remission phase of the RR- and SP-MS. Our observations of increased Ins and Ins/tCr concentration in the non-enhancing WM-lesions are consistent with histopathological and imaging studies showing astrocyte activation in intermediate and late stage demyelinated lesions [29, 30, 34, 35]. Thus the exploration of WM lesions using MRS may reflect more sensitively than Gd subtle ongoing lesional pathology independent of high level peripheral cell activation. [28, 33].

When we investigated the association between metabolite level and their ratios in NAWM and disability, we found some correlations whereas differences between the metabolites and disease course (RR vs SP) were found. tNAA level and tNAA/tCr ratio showed correlation with disability only in RRMS. These results are not surprising and are in line with previous MRS-studies reported a stronger correlation of tNAA/tCr and EDSS in patients with mild disability and short disease duration than in patients with more disability [36, 37]. In patients with primary progressive disease even no correlation of metabolites with EDSS was found [38]. Important findings of our study are the correlation of Ins/tNAA ratio with disability in patients with RRMS. This effect was not seen in patients with SPMS suggesting that gliosis may be a pathological process of clinical relevance in the relapsing forms of MS and is less present in the progressive form of the disease. In contrast, correlation of Cho with EDSS was only found in SPMS. Our results are in agreement with histopathological evidence of less pronounced inflammation in the NAWM in the progressive stage of the disease [28, 29]. These findings suggest that membrane metabolism associated with inflammatory demyelination/remyelination process is responsible for disability in the progressive form of the disease. Our hypothesis is supported by the histopathological concept of slowly expanding demyelination in SPMS [39] and MRS studies demonstrating that Cho and Cho/NAA ratio may differentiate progressive forms of MS from RRMS [16, 17].

In conclusion, metabolites concentrations measured by MRS may help to understand different immunopathogenic mechanisms that contribute to the complex pathology of MS [2, 40]. Our results of no significant difference in tNAA level between the groups suggest that axonal loss may be less pronounced than suspected during the advanced course of the disease in patients with RR- and SPMS. However, our results of tNAA decline in the NAWM in SPMS over the observation period of two years indicate ongoing diffuse axonal damage in the progressive form of the disease. The correlation of metabolites and their ratios with disability highlight the importance of reactive astrogliosis on disability in RRMS whereas the demyelination seems to be an important pathological correlate of clinical progression in SPMS.

## Supporting Information

S1 File Ethic MRS. (PDF)

S1 Table Trend Statement checklist. (PDF)
