# Supplementary material for: Brain Metabolite Changes in Patients with Relapsing-Remitting and Secondary Progressive Multiple Sclerosis: A Two-Year Follow-Up Study
Source: PLoS One. 2016 Sep 16;11(9):e0162583. doi: 10.1371/journal.pone.0162583 (PMC5026363; doi:10.1371/journal.pone.0162583)
Supplement: S1 File — (PDF) [file pone.0162583.s001.pdf]

Antrag an die Ethik-Kommission der Universität Göttingen zur Durchführung einer wissenschaftlichen Studie mit dem Titel „Untersuchung des mittels MR-Spektroskopie messbaren axonalen Schadens bei Multipler Sklerose im Verlauf von 2 Jahren“

Projektleiterin der Neurologischen Klinik: Dr. med. Muriel Sättler
Stellvertretende Projektleiterin der Neurologischen Klinik: Dr. med. Ricarda Diem
Projektleiter der MR-Forschung in der Neurologie und Psychiatrie:
Dr. rer. nat. Gunther Helms

1. Studienziel

Es soll bei Patienten mit Multipler Sklerose der Zeitpunkt des Krankheitsverlaufs bestimmt werden, an dem die Zunahme des axonalen Schadens am größten ist. Die Ergebnisse dieser Studie sollen zur Planung zukünftiger neuroprotektiver Therapiekonzepte als Ergänzung der bisherigen, anti-inflammatorischen Behandlungen dienen.

2. Wissenschaftlicher Hintergrund

Die Multiple Sklerose (MS) ist eine chronisch entzündliche Erkrankung des Zentralnervensystems, die in Deutschland mit einer Prävalenz von ca. 1:1000 auftritt und vorwiegend junge Menschen betrifft (Poser, 1994). Der typische klinische Verlauf der MS ist zunächst schubförmig-remittierend. In späteren Stadien kommt es häufig zu einem Übergang in eine sekundär chronisch progrediente Form. Neuropathologisch ist diese Erkrankung durch fokale Entzündungsherde in Gehirn und Rückenmark mit Verlust der Myelinscheiden gekennzeichnet (Prineas and Wright, 1978; Storch and Lassmann, 1997). In den letzten Jahren ist jedoch das Interesse an der zusätzlich auftretenden axonalen und neuronalen Schädigung wiedererwacht. Das Ausmaß der axonalen und neuronalen Pathologie korreliert deutlich mit den bleibenden Behinderungen der Patienten (De Stefano et al., 1998; Trapp et al., 1999). Derzeit werden verschiedene bildgebende Techniken zum Nachweis axonaler Schädigung bei MS-Patienten eingesetzt. Verglichen mit den konventionellen MRT-Techniken (T1- und T2-gewichtete Sequenzen) ist die Reduktion der N-acetyl-aspartat (NAA)-Spiegel in der MR-Spektroskopie (MRS) deutlich spezifischer für das Ausmaß des axonalen Schadens in Gehirn und Rückenmark (Birken and Oldendorf, 1989). Die NAA-Werte zeigen eine gute

Korrelation sowohl mit dem in histopathologischen Untersuchungen nachweisbaren axonalen Schaden als auch mit den klinischen Defiziten der MS-Patienten (De Stefano et al., 1998; Bitsch et al., 1999). Es wurde beobachtet, dass der mittels MRS messbare NAA-Verlust mit dem Fortschreiten der Erkrankung stetig zunimmt (Fu et al., 1998). Jedoch sind weder in besonders frühen schubförmigen Stadien noch in einer späten, ausgebrannten Phase des sekundär chronisch progredienten Verlaufs deutliche Änderungen der NAA-Werte messbar (Fu et al., 1998; Brex et al., 1999; Ruiz-Pena et al., 2004). Der genaue Zeitpunkt, an dem das Ausmaß des axonalen Schadens bei MS Patienten am schnellsten zunimmt, ist bislang nicht bekannt und soll deshalb im Rahmen dieser Studie ermittelt werden.

Abhängig davon, ob bei einem schubförmigen Verlauf mit bleibenden Residuen oder bei sekundär chronisch progredienter MS der neu auftretende axonale Schaden größer ist, sollen im Anschluss an diese Studie neuroprotektive Therapie-Ansätze für MS-Patienten geplant werden, die zuvor an Tiermodellen der MS auf ihre Wirksamkeit hin untersucht wurden. Die gängigen Behandlungskonzepte bei MS richten sich alle gegen den entzündlichen Aspekt der Erkrankung. Der neuronale und axonale Schaden wird in den etablierten Therapien bisher nicht berücksichtigt, obwohl er für die chronische Behinderung der Patienten ganz entscheidend ist.

3. Studiendesign/-durchführung

Im Rahmen der geplanten Studie möchten wir die in der MS-Ambulanz der Neurologischen Klinik eng angebundenen und bezüglich ihrer klinischen Parameter gut charakterisierten Patienten einer MRS unterziehen, um so eine spezifische Darstellung des axonalen und neuronalen Schadens zu erhalten. Ziel dieser Studie ist es, zu ermitteln, in welcher Phase der MS die Zunahme des mittels MRS messbaren axonalen und neuronalen Schadens am größten ist. Es ist geplant, zwei verschiedene Patientengruppen im Verlauf wiederholt zu untersuchen: Einerseits 20 Patienten mit einem schubförmigen Verlauf, die in der klinischen Untersuchung deutliche Residuen aufweisen. Andererseits sollen ebenfalls 20 Patienten untersucht werden, die seit weniger als 2 Jahren einen sekundär chronisch progredienten Krankheitsverlauf zeigen und hierbei eine Krankheitsaktivität mit klinisch weiterhin nachweisbarer Verschlechterung aufweisen. In beiden Gruppen sollen klinische (EDSS, MSFC) und MRS-Verlaufsuntersuchungen über 2 Jahre durchgeführt werden, im Abstand von jeweils ca. 6 Monaten. Um akut entzündliche Veränderungen auszuschließen, soll einerseits ein klinisch nachweisbarer Schub bei

der jeweiligen Untersuchung mindestens 6 Wochen zurückliegen. Zusätzlich sollen bei den MS Patienten akut entzündliche Läsionen durch Kontrastmittelgabe während der MRT-Untersuchung detektiert und ggf. von der MRS ausgeschlossen werden. Bei der gesunden Kontrollgruppe wird auf die Kontrastmittelgabe verzichtet, da bei unauffälligen nativen MRT-Bildern keine entzündliche Veränderungen zu erwarten sind, und dies keinen messbaren Einfluss auf die Ergebnisse der Studie hat. Die im Rahmen dieser Studie durchgeführten MRTs werden die sonst in diesem Zeitraum klinisch indizierten MRT-Untersuchungen ersetzen. Im Rahmen der Studie ist bei beiden Patientengruppen sowohl eine MRS von zwei Bereichen des sog. „normal appearing white matter“ (NAWM, gemäß T2-gewichteter MRT makroskopisch nicht befallene weiße Substanz) als auch von zwei chronischen MS-Läsionen geplant. Die Ergebnisse der MRS sollen durch Daten zu Substanzverlust und Demyelinisierung ergänzt werden. Die erhobenen Daten sollen auf eine Korrelation mit den klinischen Befunden hin überprüft werden. Die Erkrankungsphase, in der der neu auftretende axonale Schaden am größten ist, soll bestimmt werden. Durch einen Vergleich der MRS-Daten mit den Ergebnissen zu Demyelinisierung und Substanzverlust sollen die einzelnen MRT-Parameter zusätzlich auf ihre prognostische Wertigkeit für die Zunahme des axonalen Schadens getestet werden. Anschließend ist ein Vergleich der Patienten-Daten mit den bei gesunden Probanden erhobenen Werten vorgesehen.

Für die MRS soll das folgende Untersuchungsprotokoll verwendet werden:

1)	Sag FLASH, 1.26 mm/pixel isotropische Auflösung	ca. 6 min
2)	Sag FLASH, 1.26 mm/pixel isotropische Auflösung	ca. 6 min
3)	Sag MT-FLASH, 1.26 mm/pixel isotropische Auflösung	ca. 6 min
4)	Ax T2, TSE, 3 mm Schichtdicke	ca. 4 min
5)	Ax T1, 6 mm Schichtdicke, nativ	ca. 2 min
6)	Kontrastmittel-Gabe, „single dose“ 0,1 mmol Gd^{++}/kg KG	
7)	Ax 3D FLAIR mit 1.26 mm isotropischer Auflösung	ca. 15 min
8)	Ax T1, 6 mm, nach KM	ca. 2 min
9)	STEAM-MRS	4x ca. 7 min
	2 Volumina in NAWM	
	2 Volumina in ausgewählten Läsionen	

Bei der gesunden Kontrollgruppe entfallen die Punkte 6 und 8, da auf eine Kontrastmittelgabe verzichtet wird.

Aus den FLASH-Bilddatensätzen werden parametrische Bilder errechnet, die Veränderungen in T1 (zellulärer und axonaler Verlust) und MT (Myelinverlust) widerspiegeln. Der Zeitbedarf für eine Untersuchung beträgt inklusive von Justage- und Berechungszeiten ca. 40 min für den Bildgebungs- und 40 min für den Spektroskopieteil, also insgesamt ca. 1h 20 min. Diese Studie soll an dem 3 Tesla-Gerät durchgeführt werden, weil nur durch die mit diesem Gerät zu erreichende besonders hohe Auflösung eine zuverlässige Darstellung und Abgrenzung der Läsionslast von NAWM möglich sein wird. Sämtliche Untersuchungen, die im Rahmen dieser Studie geplant sind, werden ambulant durchgeführt werden.

Prüfprotokoll:

Die gesunden Probanden sollen nach dem folgenden Schema evaluiert werden:

Einschlußkriterien: Anamnese und klinische Untersuchung ohne Hinweis auf eine bestehende oder vorangegangene ZNS-Erkrankung, keine Kontraindikation zur Durchführung von MRT-Untersuchungen (kein Herzschrittmacher, keine implantierten Metallteile, Bereitschaft zu wiederholten MRT-Untersuchungen im Verlauf von 2 Jahren

Parameter: in den Monaten 0 (Einschlußdatum), 6, 12, 18 und 24 sollen bei gesunden Probanden die folgenden Parameter in der dargestellten Reihenfolge untersucht werden:

- Alter, Geschlecht, Körpergröße, Gewicht
- Neurologische Ausfälle nach Einschätzung des Probanden
- Anamnestischer Ausschluss einer Medikation mit Einfluss auf das ZNS
- EDSS (Expanded disability status scale, eine für MS-Patienten entwickelte klinisch-neurologische Untersuchung)
- Ausschluss des Vorliegens von ZNS-Läsionen durch neuroradiologischen Befund auf der Basis von T1- und T2-gew. MRT (Standardparameter T1: TR = 600 ms, TE = 13 ms; T2: TR = 3000 ms, TE = 100 ms)
- Bestimmung von T1- und MTR Karten (magnetization transfer ratio (MTR), als Surrogatmarker für die Myelinisierung).
- MRS-Messung der axonalen Dichte in 4 Volumina in standardisierten Regionen mit hoher Wahrscheinlichkeit für das Auftreten, bzw. Ausbleiben von

Läsionen (paraventrikulär am Vorder- und Hinterhorn, frontal und parietal im zentralen Marklager)

- Bestimmung von MTR und T1 in 4 Volumina, die mit der MRS kongruent sind.

<u>Einschlußkriterien bei MS-Patienten:</u>

Gesicherte Diagnose Multiple Sklerose, entweder schubförmige Verlaufsform mit bleibenden Residuen oder sekundär chronisch progredienter Verlauf seit maximal 3 Jahren, Vorkommen von „MRS-geeigneten“ Läsionen. Alter 18-60 Jahre.

<u>Parameter:</u> in den Monaten 0 (Einschlußdatum), 6, 12, 18 und 24 sollen in beiden Patienten-Gruppen die folgenden Parameter in der dargestellten Reihenfolge untersucht werden:

- Alter, Geschlecht, Körpergröße, Gewicht
- Dauer der Erkrankung
- Verlaufstyp, Dauer dieser Verlaufsform
- Datum des letzten Schubes (Verschieben der Untersuchung, falls weniger als 6 Wochen zurückliegend)
- Veränderung der Beschwerden in den letzten 6 Monaten (subjektive Einschätzung des Patienten)
- Aktuelle Therapie, Dauer dieser Therapie
- EDSS
- MSFC (multiple sclerosis functional composite, bestehend aus einer Gehstrecken-Messung, einem Geschicklichkeitstest (9 hole pack test) und einem Aufmerksamkeits-Gedächtnistest)
- Darstellung der ZNS-Läsionen durch neuroradiologischen Befund auf der Basis von T1- und T2-gew. MRT (Standardparameter T1: TR = 600 ms, TE = 13 ms; T2: TR = 3000 ms, TE = 100 ms)
- Nachweis von Gd-aufnehmender Läsionen in T1
- Bestimmung von T1- und MTR Karten, als Surrogatmarker für die Myelinisierung
- MRS-Messung der axonalen Dichte in 2 Volumina NAWM und in 2 Läsionen mit typischer Lokalisation (paraventrikulär am Vorder- und Hinterhorn, frontal und parietal im zentralen Marklager)
- Bestimmung von MTR und T1 in 4 Volumina, die mit der MRS kongruent sind.

4. Patienten-/Probandenrekrutierung

Im Rahmen der geplanten Studie sollen MS-Patienten mit einem schubförmigen oder sekundär chronisch progredienten Verlauf mittels MRS wiederholt untersucht werden, um den Zeitpunkt der maximalen Zunahme des axonalen Schadens zu bestimmen. Bei den einzuschließende Patienten soll die Diagnose MS entsprechend der Kriterien nach McDonalds gesichert sein (McDonalds et al., 2001), die als aktueller Standard in der diagnostischen Einstufung der MS gelten. Es sollen Patienten ausgewählt werden, die entweder einen schubförmigen Verlauf mit bleibenden Residuen aufweisen oder seit höchstens 2 Jahren eine sekundär chronisch progrediente Verschlechterung zeigen. Es sollen nur Patienten in die Studie eingeschlossen werden, die eine immunmodulatorische Therapie (Avonex, Betaferon, Copaxone oder Rebif) oder keine medikamentöse Behandlung erhalten. Zum Vergleich sollen zusätzlich 20 gesunde Kontrollen untersucht werden.

Die gesunde Kontrollgruppe wird nach demographischen Merkmalen (Alter, Geschlecht, höchster Bildungsabschluss) gematcht werden, um einen Bias auszuschließen. Da beide Patientengruppen mit den gesunden Kontrollen verglichen werden sollen, wird eine vollkommene Übereinstimmung der demographischen Daten jedoch wahrscheinlich nicht möglich sein. Leider stehen keine finanziellen Mittel zur Verfügung stehen, um die Probanden und Patienten für den ihnen entstehenden Aufwand zu entschädigen.

5. Biometrie

Die statistische Auswertung soll durch die Projektleiter der Neurologie (Dr. M. Sättler) und des Forschungs-MR (Dr. G. Helms) erfolgen. Das Hauptzielkriterium dieser wissenschaftlichen Studie ist die Änderung des NAA-Wertes im Verlauf von 2 Jahren im Vergleich zu dem Ausgangswert. Von Interesse ist insbesondere die Korrelation des NAA-Verlustes mit den klinischen Defiziten bestimmt durch EDSS und MSFC. Nebenzielkriterien in der geplanten Studie werden die jeweiligen MRS-Änderungen nach 6, 12 und 18 Monaten sein. Weiterhin soll analysiert werden, ob die ursprünglichen mittels MRS ermittelten Werte eine prognostische Aussagefähigkeit in Bezug auf die zukünftige Zunahme des axonalen Schadens bei den jeweiligen Patienten haben. Durch die Bildparameter T1 und MTR sollen Kofaktoren erfasst werden, die möglicherweise von Einfluss auf die prognostische Aussagefähigkeit sind. Die Kontrollgruppe gesunder Probanden liefert den NAA-Normalwert sowie ein

Maß für die Scan-to-scan-Varianz. Umgekehrt sichern die Probandenuntersuchungen die longitudinale Konsistenz der Ergebnisse für den unwahrscheinlichen Fall eines gerätebedingten systematischen Einflusses.

Eine Zwischenanalyse der MRS-Daten ist vorgesehen, wenn alle Patienten/Probanden die Verlaufsuntersuchungen nach 12 Monaten abgeschlossen haben. Sollte sich hierbei ein eindeutiger Unterschied zwischen beiden Patientengruppen zeigen, ist ein vorzeitiger Studienabbruch geplant, um die Patienten keiner unnötigen Belastung zu unterziehen.

5.1) Biometrisches Design

Im Rahmen dieser Studie sollen 3 Parallelgruppen untersucht werden: gesunde Probanden, MS-Patienten mit schubförmigem Verlauf und MS-Patienten mit sekundär chronisch progredientem Verlauf. Um einen Bias auszuschließen sollen dabei die gesunden Probanden bestmöglich nach demographischen Merkmalen (Alter, Geschlecht und höchster Bildungsstand) zu den beiden Patientengruppen gematcht werden. Da beide Patientengruppen mit den gesunden Kontrollen verglichen werden sollen, wird eine vollkommene Übereinstimmung der demographischen Daten wahrscheinlich nicht möglich sein.

5.2) Stichprobenplanung

Es ist geplant, insgesamt 60 Probanden zu untersuchen, die sich gleichmäßig auf die unter 5.1 genannten 3 Gruppen verteilen. Durch den Vergleich der MS-Patienten mit gesunden Probanden verbessert sich die Reliabilität, da die für diese Studie vorgesehenen MRT-Sequenzen bisher nicht für Routine-Untersuchungen etabliert sind. Sie wurden lediglich im Rahmen einzelner klinischer Studien getestet. Bedingt durch die aufwendigen MRT-Untersuchungen ist die Anzahl der Probanden, die untersucht werden können, stark eingeschränkt. Durch den Nutzerausschuss des Forschungs-MR wurde die Fallzahl von 20 Probanden pro Gruppe genehmigt und für die Untersuchung der geplanten Zielkriterien als sinnvoll und ausreichend eingeschätzt.

5.3) Datenerfassung und Auswertung

Es ist vorgesehen, die im Rahmen dieser Studie erhobenen Daten einerseits in der Patientenakte, aber auch in einer elektronischen Datenbank (MSDS, Version 3.0) zu erfassen. Diese Datenbank ist in der MS-Ambulanz der Neurologischen Klinik bereits

etabliert. Die personenbezogenen Daten der gesunden Probanden werden nur zu Studienzwecken erfasst, sie werden in einer separaten Datenbank, die in ihrem Aufbau der Datenbank der MS-Ambulanz entspricht, eingegeben werden und zusätzlich in einer für diese Studie angelegten Akte notiert werden. Die Studien-Akten der gesunden Probanden werden in einem verschlossenen Schrank in der MS-Ambulanz verwahrt werden. Zur Sicherung der Datenqualität wird in regelmäßigen Abständen ein Abgleich der mit beiden Methoden erfassten Daten durchgeführt werden. In die elektronischen Datenbanken integriert ist auch ein Auswertprogramm. Hiermit sollen die Daten über den bisherigen Krankheitsverlauf der Patienten und die aktuellen Symptome (EDSS, MSFC) bearbeitet werden, um sie anschließend in anonymisierter Form statistisch auswerten zu können.

5.4) Statistische Methoden

Bei der biometrischen Auswertung ist es vorgesehen, die einzelnen Daten zunächst graphisch darzustellen, beispielsweise in Form einer Punktewolke oder eines Boxplots. Basierend auf den hierbei resultierenden Ergebnissen soll anschließend die weitere statistische Auswertung geplant werden. Ggf. wird hierfür die Unterstützung durch die Abteilung Medizinische Statistik erbeten werden.

6. Archivierung und Datenschutz

Personenbezogene Daten, die im Rahmen dieser Studie erhoben werden, werden vom Leiter der Studie, den Prüfärzten und allen anderen an der Durchführung dieser Studie beteiligten Personen nicht weitergegeben. Eine Zuordnung der persönlichen Daten zu den Studien-Daten werden nur die Prüfärzte vornehmen. Die erfassten personenbezogenen Daten sind bei den MS Patienten zum Zwecke der Vertragsdurchführung notwendig, da die Untersuchungen im Rahmen dieser Studie sowohl eine klinisch-neurologische Untersuchung im Rahmen der MS-Ambulanz ersetzen als auch eine MRT-Verlaufskontrolle. In der gesunden Kontrollgruppe werden die personenbezogenen Daten nur zur Durchführung der Studie erhoben. Die im Rahmen dieser Studie erhobenen personenbezogenen Daten und auch die Auflösung, welche in die Studie eingegangenen Daten von welchem Patienten stammen, werden in elektronischer Form in einem Passwort geschützten Computer bzw. als Ausdruck in einem abgeschlossenen Schrank aufgehoben werden. Zugang zu dem Computer und dem verschlossenen Schrank haben nur die Prüfärzte. Nach

Abschluss der Studie werden die Prüfungsunterlagen gemäß den Bestimmungen des Datenschutzes verwaltet und archiviert.

7. Literatur

Birken DL, Oldendorf WH (1989) N-acetyl-L-aspartic acid: a literature review of a compound prominent in 1H-NMR spectroscopic studies of brain. Neurosci Biobehav Rev 13:23-31.

Bitsch A, Bruhn H, Vougioukas V, Stringaris A, Lassmann H, Frahm J, Brück W (1999) Inflammatory CNS demyelination: histopathologic correlation with in vivo quantitative proton MR spectroscopy. AJNR Am J Neuroradiol 20:1619-1627.

Brex PA, Gomez-Anson B, Parker GJ, Molyneux PD, Miszkiel KA, Barker GJ, MacManus DG, Davie CA, Plant GT, Miller DH (1999) Proton MR spectroscopy in clinically isolated syndromes suggestive of multiple sclerosis. J Neurol Sci 166:16-22.

De Stefano N, Matthews PM, Fu L, Narayanan S, Stanley J, Francis GS, Antel JP, Arnold DL (1998) Axonal damage correlates with disability in patients with relapsing-remitting multiple sclerosis. Results of a longitudinal magnetic resonance spectroscopy study. Brain 121 (Pt 8):1469-1477.

Fu L, Matthews PM, De Stefano N, Worsley KJ, Narayanan S, Francis GS, Antel JP, Wolfson C, Arnold DL (1998) Imaging axonal damage of normal-appearing white matter in multiple sclerosis. Brain 121 (Pt 1):103-113.

McDonald WI, Compston A, Edan G, Goodkin D, Hartung HP, Lublin FD, McFarland HF, Paty DW, Polman CH, Reingold SC, Sandberg-Wollheim M, Sibley W, Thompson A, van den Noort S, Weinshenker BY, Wolinsky JS (2001) Recommended diagnostic criteria for multiple sclerosis: guidelines from the International Panel on the diagnosis of multiple sclerosis. Ann Neurol 50: 121-127.

Poser CM (1994) The epidemiology of multiple sclerosis: a general overview. Ann Neurol 36 Suppl 2:S180-193.

Prineas JW, Wright RG (1978) Macrophages, lymphocytes, and plasma cells in the perivascular compartment in chronic multiple sclerosis. Lab Invest 38:409-421.

Ruiz-Pena JL, Pinero P, Sellers G, Argente J, Casado A, Foronda J, Ucles A, Izquierdo G (2004) Magnetic resonance spectroscopy of normal appearing white matter in early relapsing-remitting multiple sclerosis: correlations between disability and spectroscopy. BMC Neurol 4:8.

Storch M, Lassmann H (1997) Pathology and pathogenesis of demyelinating diseases. Curr Opin Neurol 10:186-192.

Trapp BD, Ransohoff R, Rudick R (1999) Axonal pathology in multiple sclerosis: relationship to neurologic disability. Curr Opin Neurol 12:295-302.

8. Unterschriften der Prüfärzte und der Abteilungsdirektoren

8.1) Prüfärzte

Dr. Ricarda Diem	....
	Datum, Unterschrift
Dr. Kai Kallenberg	....
	Datum, Unterschrift
Dr. Antje Kuhnert	....
	Datum, Unterschrift
Dr. Katharina Maier	....
	Datum, Unterschrift
Dr. Muriel Sättler	....
	Datum, Unterschrift

8.2) weitere an der Studie beteiligte Personen

Dr. Gunther Helms	....
	Datum, Unterschrift

8.3) Leiter der beteiligten Einrichtungen

Prof. Dr. Mathias Bähr	....
	Datum, Unterschrift
Dr. Peter Dechent	....
	Datum, Unterschrift
Prof. Dr. Michael Knauth	....
	Datum, Unterschrift

9. Patienteninformation und Einverständniserklärung

Patienteninformation zu der wissenschaftlichen Studie „Untersuchung des mittels MR-Spektroskopie messbaren axonalen Schadens bei Multipler Sklerose im Verlauf von 2 Jahren“

Dr. med. Muriel Sättler, Tel. 0551/398484, Neurologische Klinik
Universitätsklinik Göttingen, Robert-Koch-Str. 40, 37075 Göttingen

Sehr geehrte Patientin, sehr geehrter Patient,

bei Ihnen besteht eine diagnostisch gesicherte Multiple Sklerose. Dies ist eine Erkrankung, die mit einer chronischen Entzündungsreaktion in Gehirn und/oder Rückenmark einhergeht. Der primäre Angriffspunkt dieser Entzündung ist die Isolierschicht der Nervenfasern (Myelinscheiden). Man weiß jedoch seit einigen Jahren, dass auch die Nervenzellen selbst geschädigt werden können und dass dies insbesondere die Ursache bleibender Behinderungen ist. In unserer Studie möchten wir mittels moderner Untersuchungstechniken der Magnet-Resonanz-Tomographie (MRT) das Ausmaß des Nervenzellschadens im Verlauf von 2 Jahren darstellen. Für diese Messungen sind MRT-Untersuchungen mit einer Dauer von jeweils 1 Stunde und 20 Minuten notwendig. Wir möchten diese Untersuchungen fünfmal im Abstand von je 6 Monate bei Ihnen durchführen, um den Verlauf der Nervenzellschädigung zu prüfen. Zusätzlich werden wir jeweils eine detaillierte klinisch-neurologische Untersuchung bei Ihnen durchführen, wie Sie dies bereits aus der MS-Ambulanz kennen. Diese Studie werden wir bei insgesamt 60 Personen durchführen, die alle in der Neurologischen Klinik der Universitätsklinik Göttingen betreut werden
Wir bitten Sie, an dieser Studie teilzunehmen und uns so zu helfen, Ihre Erkrankung und deren Verlauf besser zu verstehen. Da es sich um eine rein wissenschaftliche Studie handelt, ist ein unmittelbarer Nutzen für Sie selbst nicht zu erwarten. Die durch die Studie gewonnenen Erkenntnisse werden uns jedoch bei der künftigen Entwicklung und dem Einsatz neuer Medikamente helfen. Sollten Sie sich gegen die Durchführung dieser Untersuchungen entscheiden oder auch Ihre Einwilligung (ohne Angabe von Gründen) zurückziehen, wird dies selbstverständlich keinerlei Nachteile bezüglich der weiteren Behandlung mit sich bringen. Ihre Teilnahme an dieser Studie wird im Rahmen der ärztlichen Schweigepflicht vertraulich behandelt.

Einwilligungserklärung zum Datenschutz ~~(Arzneimittelgesetz)~~

Bei klinischen Studien werden persönliche Daten und medizinische Befunde über Sie erhoben. Die Erhebung, Weitergabe, Speicherung und Auswertung dieser Angaben über ihre Gesundheit erfolgt nach gesetzlichen Bestimmungen und setzt vor Teilnahme an der klinischen Studie folgende freiwillige Einwilligung voraus, d.h. ohne die nachfolgende Einwilligung können Sie nicht an der klinischen Studie teilnehmen.
1.) Ich erkläre mich damit einverstanden, dass im Rahmen dieser klinischen Studie erhobene Daten/Angaben über meine Gesundheit auf Fragebögen und elektronischen Datenträgern aufgezeichnet und ohne Namensnennung (pseudonymisiert) weitergegeben werden an die zuständige Ethik-Kommission zur Überprüfung der ordnungsgemäßen Durchführung der Studie und zur Bewertung von Studienergebnissen und unerwünschter Ereignisse.

2.) Die Einwilligung zur Erhebung und Verarbeitung der Angaben über meine Gesundheit ist unwiderruflich. Ich bin bereits darüber aufgeklärt worden, dass ich jederzeit die Teilnahme an der klinischen Studie beenden kann. Im Fall dieses Widerrufs erkläre ich mich damit einverstanden, dass die bis zu diesem Zeitpunkt gespeicherten Daten ohne Namensnennung weiterhin verwendet werden dürfen, soweit dies erforderlich ist, um sicherzustellen, dass schutzwürdige Interessen der betroffenen Person nicht beeinträchtigt werden.

Dr. Muriel Sättler
Neurologische Klinik
Tel.: 0551/398484

Uniklinikum Göttingen
Robert-Koch-Str. 40
37075 Göttingen

Patienteneinverständniserkärung zu der Studie „Untersuchung des mittels MR-Spektroskopie messbaren axonalen Schadens bei Multipler Sklerose im Verlauf von 2 Jahren“

Patientenaufkleber

Ich,..., wurde von meinem Arzt / meiner Ärztin vollständig über Wesen, Bedeutung und Tragweite der wissenschaftlichen Studie mit dem o.g. Titel aufgeklärt. Ich habe den Aufklärungstext gelesen und verstanden. Ich hatte die Möglichkeit, Fragen zu stellen, und habe die Antworten verstanden und akzeptiere sie.

Ich hatte ausreichend Zeit, mich zur Teilnahme an dieser Studie zu entscheiden und weiß, dass die Teilnahme freiwillig ist. Ich weiß, dass ich jederzeit und ohne Angabe von Gründen diese Zustimmung widerrufen kann, ohne dass sich dieser Entschluss nachteilig auf die spätere Behandlung durch meinen Arzt auswirken wird.

Mir ist bekannt, dass meine persönlichen Daten in verschlüsselter Form gespeichert werden.

Mit meinem Einverständnis zur Teilnahme erkläre ich gleichzeitig, dass ich mit der im Rahmen dieser klinischen Prüfung erfolgenden Aufzeichnung von Krankheitsdaten und ihrer Weitergabe zur Überprüfung an die zuständigen Überwachungsbehörden oder die zuständige Bundesoberbehörde einverstanden bin.

Ich gestatte hiermit, dass offizielle Vertreter zuständiger in- und ausländischer Behörden unter Wahrung der ihnen auferlegten Schweigepflicht Einblick in meine personenbezogenen Krankenakten nehmen, soweit mir die Einsicht in die Krankenakten zusteht.

Ich habe eine Kopie der Patienteninformation und dieser Einwilligungserkärung erhalten. Ich erkläre hiermit meine freiwillige Teilnahme an dieser wissenschaftlichen Studie.

Ort und Datum Unterschrift des Patienten

Ort und Datum Unterschrift des aufklärenden Arztes

10. Probandeninformation und Einverständniserklärung

Probandeninformation zu der wissenschaftlichen Studie „Untersuchung des mittels MR-Spektroskopie messbaren axonalen Schadens bei Multipler Sklerose im Verlauf von 2 Jahren“

Dr. med. Muriel Sättler, Tel. 0551/398484, Neurologische Klinik
Universitätsklinik Göttingen, Robert-Koch-Str. 40, 37075 Göttingen

Sehr geehrte Damen und Herren,

zur Erforschung des Krankheitsverlaufs bei Multipler Sklerose wird an der Neurologischen Klinik des Universitätsklinikums Göttingen eine wissenschaftliche Studie mit dem oben aufgeführten Titel durchgeführt. Multiple Sklerose ist eine Erkrankung, die mit einer chronischen Entzündungsreaktion in Gehirn und/oder Rückenmark einhergeht. Der primäre Angriffspunkt dieser Entzündung ist die Isolierschicht der Nervenfasern (Myelinscheiden). Man weiß jedoch seit einigen Jahren, dass auch die Nervenzellen selbst geschädigt werden können und dass dies insbesondere die Ursache bleibender Behinderungen bei den Betroffenen ist. In unserer Studie möchten wir mittels moderner Untersuchungstechniken der Magnet-Resonanz-Tomographie (MRT) das Ausmaß des Nervenzellschadens im Verlauf von 2 Jahren darstellen. Um die bei Patienten mit Multipler Sklerose erhobenen Daten auswerten zu können, benötigen wir zum Vergleich MRT-Aufnahmen von gesunden Personen. Auch bei Ihnen als Kontrollperson würden wir, wenn Sie zustimmen, an dieser Studie teilzunehmen, im Verlauf von 2 Jahren fünfmal MRT-Untersuchungen im Abstand von je 6 Monate durchführen. Diese Messungen werden jeweils 1 Stunde und 20 Minuten dauern. Zusätzlich werden wir Sie an jedem der 5 Termine nach gesundheitlichen Beschwerden und nach Medikamenten, die Sie einnehmen, befragen und eine detaillierte klinisch-neurologische Untersuchung bei Ihnen durchführen, d.h., dass Sie von einer Ärztin/einem Arzt während etwa 15 Minuten körperlich untersucht werden. Diese Studie werden wir bei insgesamt 60 Personen durchführen, die alle in der Neurologischen Klinik der Universitätsklinik Göttingen betreut werden

Wir bitten Sie, an dieser Studie teilzunehmen und uns so zu helfen, die Multiple Sklerose und ihren Verlauf besser zu verstehen. Da es sich um eine rein wissenschaftliche Studie handelt, ist ein unmittelbarer Nutzen für Sie selbst nicht zu erwarten. Die durch die Studie gewonnenen Erkenntnisse werden uns jedoch bei der

künftigen Entwicklung und dem Einsatz neuer Medikamente helfen. Sollten Sie sich gegen die Durchführung dieser Untersuchungen entscheiden oder auch Ihre Einwilligung (ohne Angabe von Gründen) zurückziehen, wird dies selbstverständlich keinerlei Nachteile mit sich bringen. Ihre Teilnahme an dieser Studie wird im Rahmen der ärztlichen Schweigepflicht vertraulich behandelt.

Einwilligungserklärung zum Datenschutz

Bei klinischen Studien werden persönliche Daten und medizinische Befunde über Sie erhoben. Die Erhebung, Weitergabe, Speicherung und Auswertung dieser Angaben über ihre Gesundheit erfolgt nach gesetzlichen Bestimmungen und setzt vor Teilnahme an der klinischen Studie folgende freiwillige Einwilligung voraus, d.h. ohne die nachfolgende Einwilligung können Sie nicht an der klinischen Studie teilnehmen.

1.) Ich erkläre mich damit einverstanden, dass im Rahmen dieser klinischen Studie erhobene Daten/Angaben über meine Gesundheit auf Fragebögen und elektronischen Datenträgern aufgezeichnet und ohne Namensnennung (pseudonymisiert) weitergegeben werden an die zuständige Ethik-Kommission zur Überprüfung der ordnungsgemäßen Durchführung der Studie und zur Bewertung von Studienergebnissen und unerwünschter Ereignisse.

2.) Die Einwilligung zur Erhebung und Verarbeitung der Angaben über meine Gesundheit ist unwiderruflich. Ich bin bereits darüber aufgeklärt worden, dass ich jederzeit die Teilnahme an der klinischen Studie beenden kann. Im Fall dieses Widerrufs erkläre ich mich damit einverstanden, dass die bis zu diesem Zeitpunkt gespeicherten Daten ohne Namensnennung weiterhin verwendet werden dürfen, soweit dies erforderlich ist, um sicherzustellen, dass schutzwürdige Interessen der betroffenen Person nicht beeinträchtigt werden.

Dr. Muriel Sättler
Neurologische Klinik
Tel.: 0551/398484

Uniklinikum Göttingen
Robert-Koch-Str. 40
37075 Göttingen

Probandeneinverständniserkärung zu der Studie „Untersuchung des mittels MR-Spektroskopie messbaren axonalen Schadens bei Multipler Sklerose im Verlauf von 2 Jahren“

Probandenaufkleber

Ich,..., wurde von meinem Arzt / meiner Ärztin vollständig über Wesen, Bedeutung und Tragweite der wissenschaftlichen Studie mit dem o.g. Titel aufgeklärt. Ich habe den Aufklärungstext gelesen und verstanden. Ich hatte die Möglichkeit, Fragen zu stellen, und habe die Antworten verstanden und akzeptiere sie.

Ich hatte ausreichend Zeit, mich zur Teilnahme an dieser Studie zu entscheiden und weiß, dass die Teilnahme freiwillig ist. Ich weiß, dass ich jederzeit und ohne Angabe von Gründen diese Zustimmung widerrufen kann, ohne dass sich dieser Entschluss für mich nachteilig auswirken wird.

Mir ist bekannt, dass meine persönlichen Daten in verschlüsselter Form gespeichert werden.

Mit meinem Einverständnis zur Teilnahme erkläre ich gleichzeitig, dass ich mit der im Rahmen dieser klinischen Prüfung erfolgenden Aufzeichnung von Krankheitsdaten und ihrer Weitergabe zur Überprüfung an die zuständigen Überwachungsbehörden oder die zuständige Bundesoberbehörde einverstanden bin.

Ich gestatte hiermit, dass offizielle Vertreter zuständiger in- und ausländischer Behörden unter Wahrung der ihnen auferlegten Schweigepflicht Einblick in meine personenbezogenen Krankenakten nehmen, soweit mir die Einsicht in die Krankenakten zusteht.

Ich habe eine Kopie der Probandeninformation und dieser Einwilligungserkärung erhalten. Ich erkläre hiermit meine freiwillige Teilnahme an dieser wissenschaftlichen Studie.

Ort und Datum | Unterschrift des Probanden

Ort und Datum | Unterschrift des aufklärenden Arztes
